# Accuracy of digitally designed endodontic posts: a micro-computed tomography analysis of intraoral scanner and extraoral scanning techniques

**DOI:** 10.1186/s12903-025-07641-4

**Published:** 2026-01-13

**Authors:** Seray Çapar, Zelal Seyfioğlu Polat, Ali Keleş

**Affiliations:** 1 Department of Prosthodontics, School of Dentistry, Dicle University, Sur, Diyarbakır, 21000 Turkey; 2 Department of Endodontics, School of Dentistry, Abant İzzet Baysal University, Bolu, Turkey

**Keywords:** 3D resin post, Digital scan, Canal measurement, Micro-Computed tomography, Additive manufacturing

## Abstract

**Background:**

The digital scanning step represents a critical stage in ensuring an accurate treatment protocol. Due to the challenges associated with intracanal scanning, such as limited direct visualization of the working field and restricted access, the selection of appropriate intracanal digitization techniques for custom post fabrication becomes essential to achieve precise dimensional accuracy.

**Purpose:**

This study evaluated the fit of three-dimensional-printed posts within root canals, using micro-computed tomography to assess the internal fit and compatibility based on the canal geometry. These canal dimensions were obtained using different digital scanning workflows.

**Materials and methods:**

Following root canal shaping, a 7-mm post-cavity preparation was made using human mandibular premolars (*N* = 35). Specimens were divided into five groups (*n* = 7) based on the digital scanning method used: direct scan via iTero Element 2, direct scan via Trios 3, indirect scan via iTero Element 2, indirect scan via Trios 3, and indirect scan via Vinyl UXD. Post designs were created using Exocad DentalCAD software and fabricated with the Asiga Max UV 3D printer employing digital light processing. Posts were printed using a resin-based material (Saremco Print Crowntec) and cemented with Panavia SA Cement Plus. Samples were scanned using a high-resolution micro-CT system (85 kV, 118 µA, voxel size 13.68 μm, 180° rotation, 2600 ms exposure). Internal canal compatibility was assessed using the resulting micro-computed tomography data.

**Results:**

Posts fabricated using indirect digital scanning workflow from the Vinyl UXD extraoral scanner demonstrated superior canal compatibility. Micro-computed tomography analysis revealed that this method produced statistically significant differences from those of the other groups (*p* < 0.05).

**Conclusions:**

Among the evaluated techniques, the extraoral indirect digital scanning workflow, involving the scanning of conventional resin impressions with an extraoral scanner, yielded the highest internal canal compatibility of the fabricated posts. These findings support the potential clinical viability of the permanent resin material used in three-dimensional printing for post fabrication.

## Clinical implications

This study provides an evaluation of the accuracy of diverse scanning techniques employed in the fabrication of custom endodontic posts to ensure optimal internal fit within root canal systems. Variability in digital scanning protocols may significantly affect the internal adaptation of posts, thereby impacting the clinical performance and long-term prognosis.

## Introduction

Root canal-treated teeth often require post placement prior to prosthetic rehabilitation to compensate for crown damage and tissue loss resulting from endodontic procedures [1–3]. The primary purpose of post systems is to reinforce the restoration, replace the lost dental structure, and provide retention for the core material. Posts function by distributing occlusal forces evenly to the radicular dentin and surrounding tissues, thereby preserving the remaining coronal tooth structure. This biomechanical support contributes to both the functional stability and the aesthetic success of the final restoration. Clinical investigations concerning the use of posts in severely damaged teeth have been long established [4].

Custom post fabrication can be performed using either the lost-wax technique or Computer-Aided Design/Computer-Aided Manufacturing (CAD–CAM) systems. The core principle of this approach is to preserve root dentin by minimizing or eliminating post space preparation, thereby maintaining thick dentin walls. This personalized strategy not only enhances the structural integrity of the tooth but also contributes significantly to the long-term success of the restoration [5].

The adoption of digital workflows in dentistry has grown rapidly, driven by advancements in intraoral scanners, dental software, and restorative materials. These technologies have become integral to daily clinical practice and enhance communication between dentists and dental technicians [6]. In CAD–CAM systems, optical impressions are captured intraorally, replacing conventional impression techniques. Instead of traditional wax modeling, restorations are digitally designed using specialized software and fabricated through automated computer-aided milling units, eliminating manual intervention [7, 8].

Digital imaging involves capturing the prepared tooth and surrounding intraoral tissues using scanning technology [9]. With the rapid advancement of optical scanning systems, digital imaging has become a critical step in ensuring the accuracy of CAD/CAM restorations. The process begins with data acquisition, producing a point cloud that represents the scanned surfaces. Then, this point cloud is converted into a continuous surface model by CAD software algorithms, a step that may introduce minor accuracy loss. Several technical factors can affect scanning precision, including the ambient lighting, software version, scanner optics, depth of field, and scanning strategy [10]. Despite potential irregularities or low-density areas in the point cloud, modern scanners use algorithmic compensation to generate accurate digital models [10]. This process, known as digital scan acquisition, eliminates the need for conventional impressions. The resulting data can be captured using various intraoral scanners [11] and transferred to CAD software for virtual restoration design [12]. In the manufacturing phase, the CAM system fabricates the restoration using subtractive, additive, or hybrid methods, fully automated via computer.

Digital technologies have become increasingly critical in restorative and endodontic procedures. Digital scanning systems are now routinely employed to capture the morphology of post spaces and facilitate the fabrication of custom endodontic posts. The accuracy of scan data is inherently influenced by the optical properties and computational design of the scanners used, including lateral resolution, axial depth of field, triangulation geometry, and reconstruction algorithms. Numerous studies demonstrate that intraoral scanners rely on sequential image-stitching processes, which can accumulate local errors into overall deviations, especially when scanning deep, narrow, or complex regions where optical penetration and surface visibility are limited [13, 14]. Conversely, extraoral laboratory scanners, which benefit from controlled lighting conditions, fixed imaging setups, and multi-camera systems, tend to generate more stable and precise datasets with significantly less geometric distortion [13, 15]. These fundamental differences in data acquisition methods result in measurable variations in the accuracy of digital reproduction of post spaces, directly affecting the fit of the final post.

In recent years, considerable progress has been made in developing noninvasive techniques for investigating the morphology of biological samples. Among these, micro-computed tomography (micro-CT) has become an essential tool for detailed analysis of root canal anatomy. This technique enables the acquisition of both qualitative and quantitative data without damaging the specimen, thereby enhancing the reliability of findings in endodontic research. Owing to its precision and versatility, micro-CT has gained widespread use in contemporary endodontic studies [16, 17].

This study aimed to evaluate the adaptation of 3D-printed posts fabricated through different digital scanning techniques and to compare the accuracy of intraoral and extraoral scanning workflows using micro-CT analysis.

The null hypothesis of this study was that there would be no significant difference in the internal adaptation of posts fabricated using different digital scanning techniques.

## Materials and methods

In all, 35 human mandibular premolars, extracted within 6 months of use primarily for orthodontic reasons and free of caries and structural loss, were selected based on similar root lengths and canal morphology. Crowns were sectioned 2 mm coronally from the enamel–cementum junction, perpendicular to the long axis, using a slow-speed handpiece and diamond discs under water cooling to prevent thermal damage. To preserve root dentin and minimize tissue loss, root canal obturation was omitted. However, canal access and morphology were assessed through shaping procedures. Root canal shaping was performed using a 0.25-mm (ISO 25) Dentac T-EndoMust (Dentac, İstanbul, Turkey) R25 reciprocating file with a VDW Reciproc Endomotor (Dentsply Sirona, Munich, Germany). Irrigation was carried out with 2% chlorhexidine solution, and canals were dried using T-Endo Paper Points. Post cavity preparation was standardized across all samples using a size 2 drill from the RelyX post system (3 M ESPE, Seefeld, Germany), creating cavities 7 mm deep and 1.6 mm in diameter.

A total of 35 teeth were divided into five experimental groups based on the canal measurement method employed in the digital workflow for the post fabrication. While the post material, production technique, and cement were consistent across all samples, grouping was determined by the digital protocol. Table 1 summarizes the sample groups and their characteristics.

**Table 1:** Grouping of the specimens

Groups	*n*	Digital Scanning Protocol	Scanner
iTero-D	7	Directly, with an intraoral scanner	iTero Element 2
iTero-ID	7	Indirectly, with an intraoral scanner	iTero Element 2
Trios-D	7	Directly, with an intraoral scanner	Trios 3
Trios-ID	7	Indirectly, with an intraoral scanner	Trios 3
Vinyl UXD	7	Indirectly, with an extraoral scanner	Vinyl UXD

In the iTero-D and Trios-D groups, post cavity dimensions were obtained via direct intra-canal scanning using the iTero Element 2 (Align Technologies, San Jose, CA) and Trios 3 (3Shape, Copenhagen, Denmark) intraoral scanners. Scanning was performed in model scan mode, and the resulting data were exported in stereolithography (STL) format and sent to the laboratory for post fabrication.

**Fig. 1:**
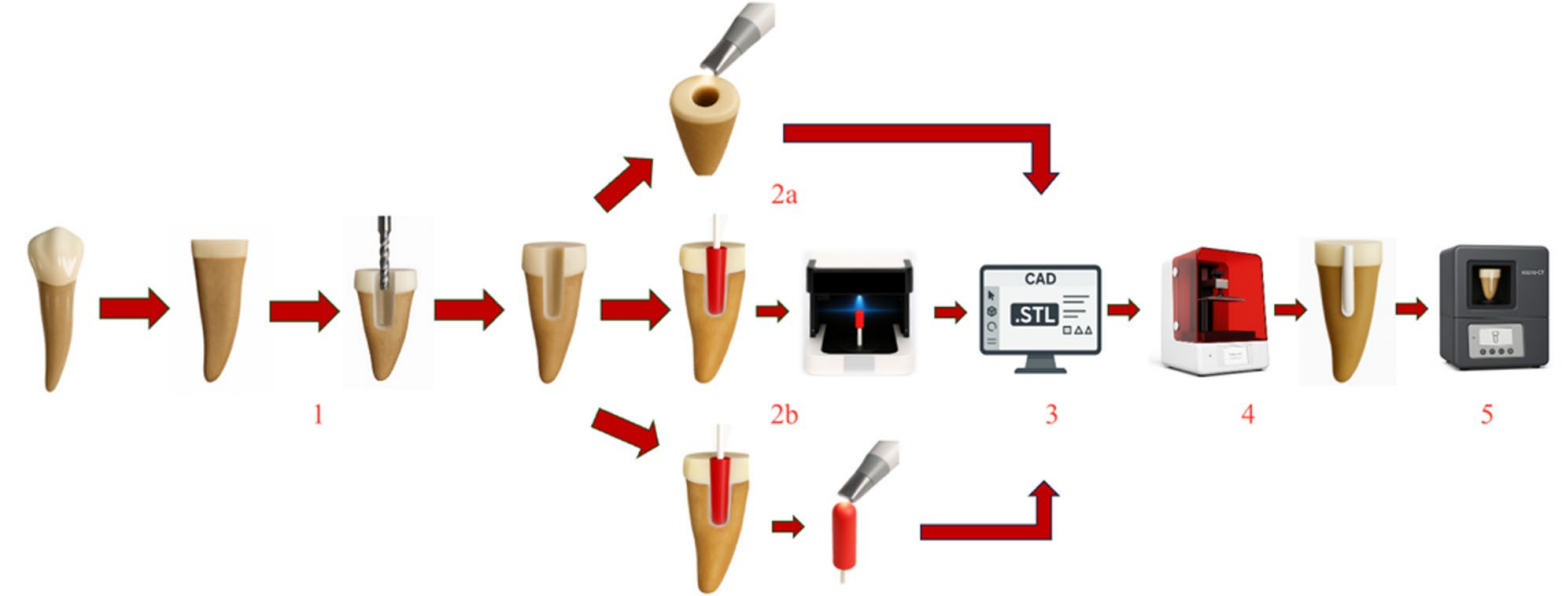
Schematic depiction of the methodological workflow delineating each phase of the experimental procedure: (1) root canal shaping succeeded by standardized post-space preparation; (2 **a-b**) digital capture of the post-space geometry utilizing intraoral or extraoral scanning techniques; (3) CAD-driven design of customized posts employing dental design software; (4) manufacturing of the posts via high-resolution DLP 3D printing with photopolymerizable resin; and (5) micro-CT imaging followed by quantitative assessment of internal fit, including measurements of post–canal wall distances

For the iTero-ID and Trios-ID groups, post cavity measurements were first obtained using Pattern Resin LS (GC Corporation, Tokyo, Japan) applied to the prepared root canals using conventional techniques. Then, the resulting resin patterns were scanned extraorally using the iTero Element 2 and Trios 3 intraoral scanners in model scan mode, with assistance from the systems’ AI-based data editors. The scan data were exported in STL format and forwarded to the laboratory for the post fabrication. For the Vinyl UXD group (Smart Optic Sensortechnik GmbH, Bochum, Germany), post cavity measurements were similarly obtained using LS Pattern Resin. These impressions were scanned extraorally using the Vinyl UXD dental model scanner, and the resulting data were also converted into STL format for digital processing. The scanning area was restricted to the occlusal surface of one tooth in all specimens. Figure 1 presents a schematic diagram that visually illustrates the methodological workflow and clarifies the sequence of experimental procedures performed in this study.

Post designs for all samples were created using Exocad DentalCAD software (Darmstadt, Germany), with the cement space set to 0 μm. The posts were fabricated using the Asiga Max UV 3D printer (Sydney, Australia), which operates via digital light processing, and printed with Saremco Print Crowntec resin (St. Gallen, Switzerland). Cementation was performed using Panavia SA Cement Plus (Kuraray, Okayama, Japan), a resin-based luting agent.

Specimens were scanned using a micro-CT system (SkyScan 1172; Bruker-microCT, Kontich, Belgium) equipped with an 11-MP camera. Scanning parameters included 85 kV voltage, 118 µA current, a pixel size of 13.68 μm, and a resolution of 2,000 × 1,330 pixels. Each sample was rotated 180° around the vertical axis with a 0.6° rotation step, 2,600 exposures, and frame averaging of 2. A 500-µm aluminum filter and a 38-µm copper filter were used during image acquisition.

Horizontal cross-sections of the samples were reconstructed using NRecon software (v.1.7.1.1; Bruker-microCT) with %45 beam-hardening correction, a smoothing factor of 2, attenuation coefficient values set between 0 and 0.16, and a ring artifact correction level of 7. Then, the reconstructed images were imported into CTAn software (v.1.18.8; Bruker-microCT) for 3D analysis. The distance between the post and the root canal wall was measured in millimeters using this software.

**Fig. 2:**
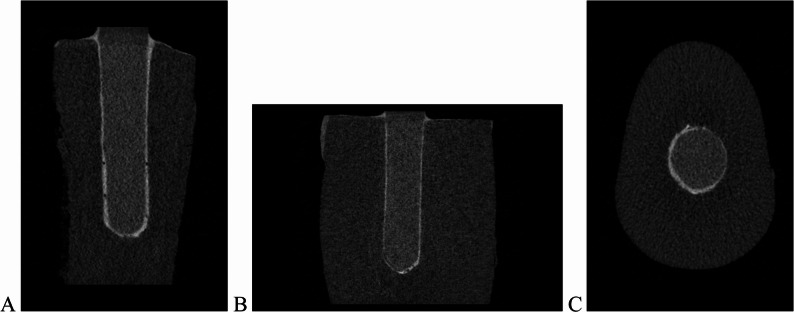
Micro-CT image of a specimen restored with a 3D-printed post. **A**, Sagittal view. **B**, Coronal view.** C**, Transaxial view A B C

**Fig. 3:**
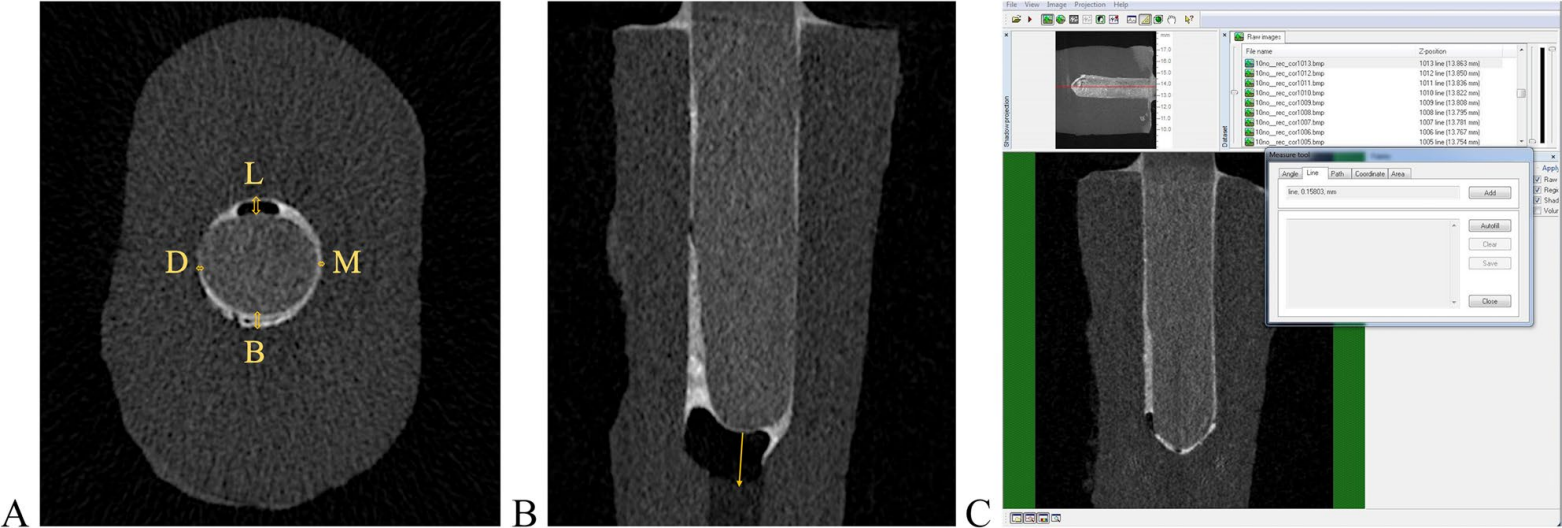
Measurement of the distances between the post and root canal. **A**, Mesial, distal, buccal, and lingual post-root canal distances on the transaxial micro-CT view.** B**, Post apex-root canal apex distance on the sagittal micro-CT section. **C**, Measurement of the distance between the post and the root canal wall using CTAn software A B C

For each sample, the root was divided into three regions: the coronal point (closest to the crown), the apical point (closest to the apex), and the midpoint between these two reference locations. Using transaxial, coronal, and sagittal views (Figs. 2A), the distance between the widest point of the post cavity and the post surface was measured at the coronal, middle, and apical levels on the buccal, lingual, distal, and mesial surfaces (Figs. 3A–C). These measurements were recorded in millimeters. Post-adaptation was evaluated through statistical analysis of the resulting data, with eight distance values obtained per sample.

In this study, the Kruskal–Wallis test, a non-parametric statistical method, was used to compare the mean values among the groups. For multiple comparisons, the Mann–Whitney U and Wilcoxon signed-rank tests were employed. Descriptive statistics and data analyses were performed using the R statistical software (version 4.1.3; The R Foundation for Statistical Computing, Vienna, Austria). Results with *P* < 0.05 were considered statistically significant. Non-parametric tests were chosen due to violations of parametric assumptions, specifically normality and homogeneity of variance, as well as the limited sample size. The Kruskal–Wallis test is appropriate for comparing independent groups under these conditions, as it evaluates the distribution of ranks rather than raw data, offering a more robust approach for datasets characterized by non-normality, outliers, or small sample sizes.

## Results

The analysis revealed several significant findings, demonstrating variations among the groups with respect to internal fit values. The tables (Tables 2 and 3, and 4) display the mean measurements in millimeters for the mesial, lingual, distal, and buccal surfaces across the apical, middle, and coronal regions, along with corresponding results from multiple comparison tests.

**Table 2:** Mean measurement values in millimeters for the mesial, lingual, distal, and buccal surfaces in the apical region, along with the results of multiple comparisons among the experimental groups

Parameters	Groups	*n*	Mean	Chi-square	Multiple comparisons *p* < 0,05
*Apical-Mesial*	iTero D	7	0,16186	*p* = 0,676	**ns**
	iTero ID	7	0,14657		
	Trios D	7	0,16057		
	Trios ID	7	0,22157		
	Vinyl UXD	7	0,13800		
*Apical-Lingual*	iTero D	7	0,10629	*p* = 0,**020**	**(2)**,**(4)**
	iTero ID	7	0,24871		**(1)**,**(3) (5)**
	Trios D	7	0,15100		**(2)**
	Trios ID	7	0,26229		**(1)**,**(5)**
	Vinyl UXD	7	0,13300		**(2)**,**(4)**
*Apical-Distal*	iTero D	7	0,17271	*p* = 0,216	**ns**
	iTero ID	7	0,19929		
	Trios D	7	0,13786		
	Trios ID	7	0,16800		
	Vinyl UXD	7	0,11700		
*Apical-Buccal*	iTero D	7	0,13800	*p* = 0,518	**ns**
	iTero ID	7	0,22129		
	Trios D	7	0,20300		
	Trios ID	7	0,20786		
	Vinyl UXD	7	0,16214		

**Table 3:** Mean measurement values in millimeters for the mesial, lingual, distal, and buccal surfaces in the middle region, along with the results of multiple comparisons among the experimental groups

Parameters	Groups	*n*	Mean	Chi-square	Multiple comparisons *p* < 0,05
*Middle-Mesial*	iTero D	7	0,08500	*p* = 0,276	**ns**
	iTero ID	7	0,13157		
	Trios D	7	0,12629		
	Trios ID	7	0,08529		
	Vinyl UXD	7	0,06629		
*Middle-Lingual*	iTero D	7	0,08929	*p* = 0,**037**	**(2)**
	iTero ID	7	0,24671		**(1)**,**(3) (4)**
	Trios D	7	0,11443		**(2)**
	Trios ID	7	0,12200		**(2)**
	Vinyl UXD	7	0,14900		**ns**
*Middle-Distal*	iTero D	7	0,11657	*p* = 0,247	**ns**
	iTero ID	7	0,12157		
	Trios D	7	0,09457		
	Trios ID	7	0,09700		
	Vinyl UXD	7	0,06957		
*Middle-Buccal*	iTero D	7	0,09443	*p* = 0,337	**ns**
	iTero ID	7	0,18443		
	Trios D	7	0,12529		
	Trios ID	7	0,15914		
	Vinyl UXD	7	0,12371		

**Table 4:** Mean measurement values in millimeters for the mesial, lingual, distal, and buccal surfaces in the coronal region, along with the results of multiple comparisons among the experimental groups

Parameters	Groups	*n*	Mean	Chi-square	Multiple comparisons *p* < 0,05
*Coronal-Mesial*	iTero D	7	0,08943	*p* = 0,181	**ns**
	iTero ID	7	0,16129		
	Trios D	7	0,09186		
	Trios ID	7	0,08957		
	Vinyl UXD	7	0,07100		
*Coronal-Lingual*	iTero D	7	0,06843	*p* = 0,**007**	**(2)**,**(3)**
	iTero ID	7	0,16443		**(1)**,**(4) (5)**
	Trios D	7	0,13143		**(1)**
	Trios ID	7	0,09000		**(2)**
	Vinyl UXD	7	0,08571		**(2)**
*Coronal-Distal*	iTero D	7	0,07629	*p* = 0,937	**ns**
	iTero ID	7	0,11457		
	Trios D	7	0,10129		
	Trios ID	7	0,08643		
	Vinyl UXD	7	0,06757		
*Coronal-Buccal*	iTero D	7	0,10100	*p* = 0,**024**	**(2)**
	iTero ID	7	0,18643		**(1)**,**(3)**,**(4)**,**(5)**
	Trios D	7	0,10914		**(2)**
	Trios ID	7	0,08000		**(2)**
	Vinyl UXD	7	0,07843		**(2)**

In the apical region, posts fabricated using indirect scanning of pattern resin with the iTero Element 2 and Trios 3 scanners (iTero-ID and Trios-ID) exhibited greater post–root canal wall distances across all surfaces (mesial, lingual, distal, and buccal) compared to the other groups. Notably, the apical lingual distances showed statistically significant differences (*P* < 0.05 unless specified otherwise) among all groups.

In the middle region, posts produced via both indirect extraoral scanning (iTero-ID, Trios-ID) and direct intraoral scanning (iTero-D, Trios-D) generally demonstrated larger post–canal wall distances than those in the other groups. Statistically significant differences were observed in the middle lingual distances across all groups, except for Group 5.

In the coronal region, the largest post–canal wall distances were observed in the iTero-ID and Trios-D groups. In contrast, the Vinyl UXD group, which utilized extraoral scanning of pattern resin, exhibited the smallest distances in this region, indicating the most precise post adaptation. Statistically significant differences were found in the coronal lingual and buccal areas across all groups.

**Fig. 4:**
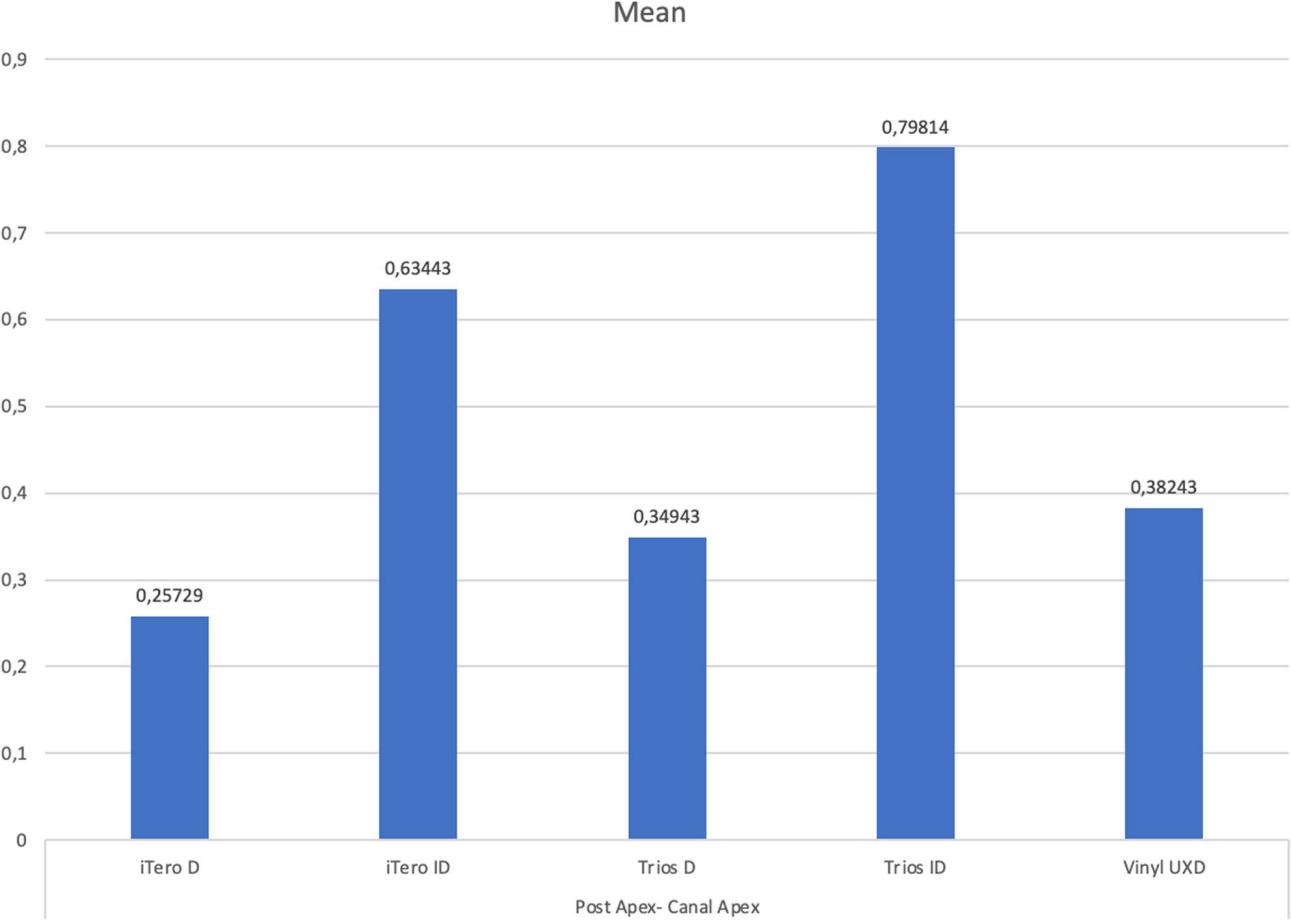
The average distances between the post apex and the root canal wall for each group

When evaluating the distance from the apex of the post to the apex of the root canal wall, the iTero-ID group showed the highest values, followed by the Trios-ID group. These findings suggest a comparatively larger cement gap at the post base in these groups. The smallest apex-to-apex distance was recorded in the iTero-D group, with the Vinyl UXD and Trios-ID groups showing similar values. However, these differences were not statistically significant (Fig. 4).

**Fig. 5:**
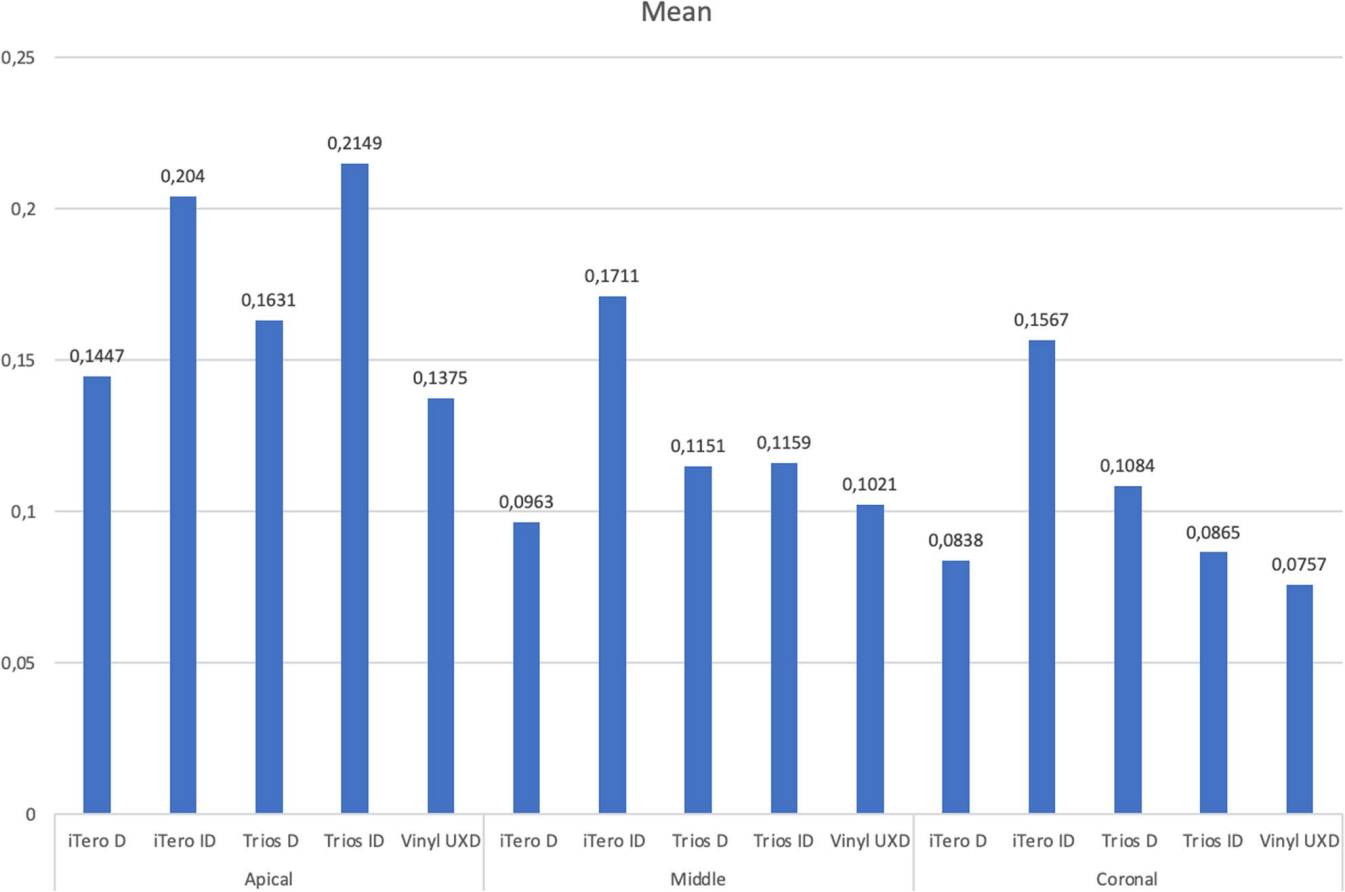
The average distances between the post and the root canal wall in the apical, middle, and coronal regions

When comparing the average post–canal wall distances across the apical, middle, and coronal regions, the Vinyl UXD group consistently demonstrated the smallest average distances. Conversely, the iTero-ID group exhibited the highest average values. While no statistically significant differences were found in the apical and middle regions, the coronal region showed significant variation among the experimental groups (Fig. 5).

**Fig. 6:**
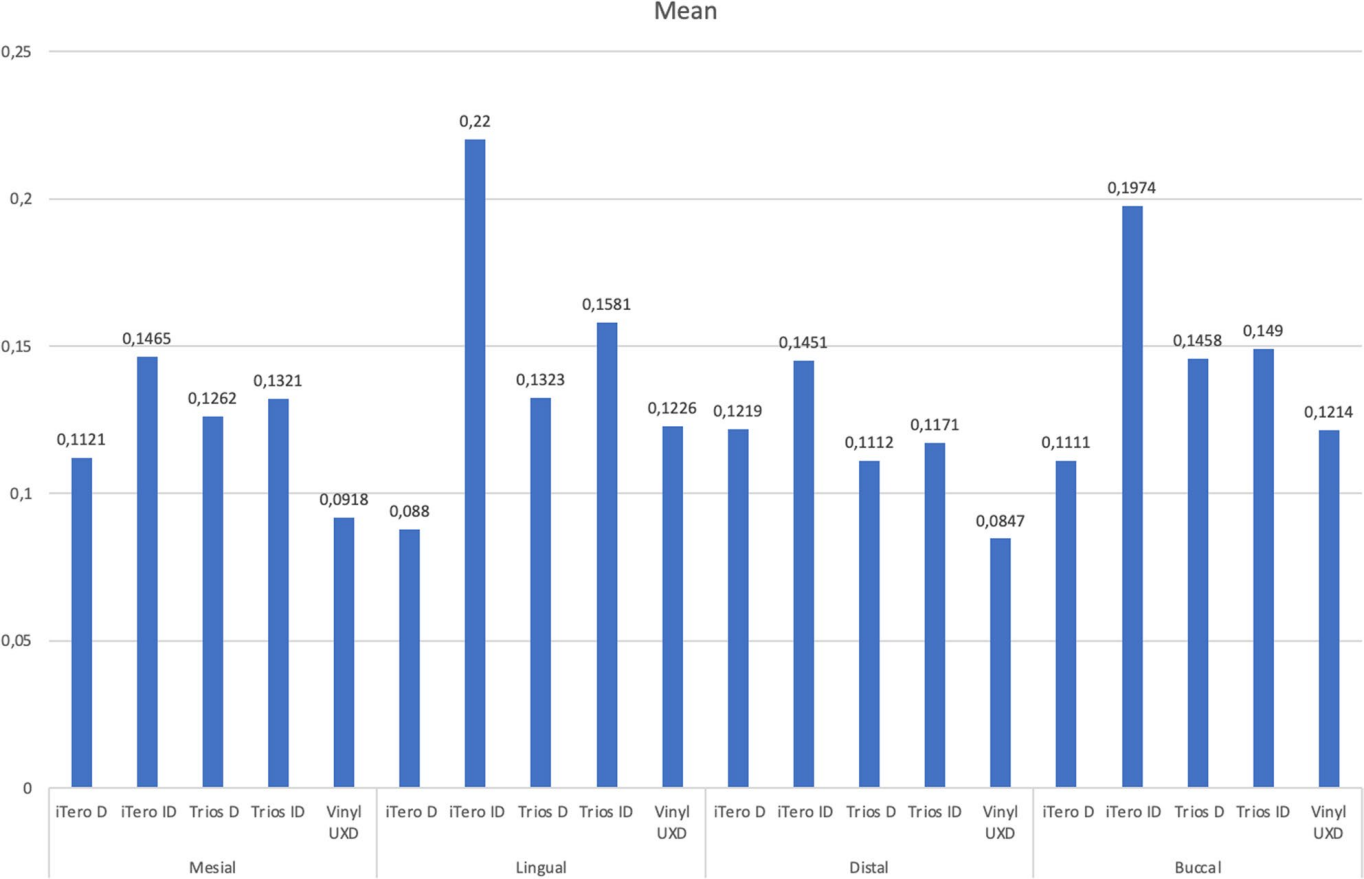
The average distances between the post and the root canal wall in the mesial, lingual, distal, and buccal regions

Analysis of average distances across the mesial, lingual, distal, and buccal surfaces revealed that the Vinyl UXD group again had the smallest values, while the iTero-ID group had the largest. Although no statistically significant differences were observed in the mesial, distal, and buccal regions, the lingual region showed significant differences across groups (Fig. 6).

## Discussion

Advancements in CAD/CAM technology have prompted extensive investigation into its application for fabricating custom posts and cores. Awad and Marghalani first introduced this approach in 2007 and subsequently raised questions that spurred further research [18, 19]. Initially, CAD/CAM workflows relied on scanning traditional plaster models; however, alternative methods have since emerged, including the scanning of silicone intraoral impressions for digital post and core fabrication [20]. Studies have demonstrated that both conventional impressions and plaster models can be digitized with high precision and reliability [21]. In 2017, Pinto et al. evaluated the fit accuracy of digital scans and conventional post impressions for post cavities with different root canal preparation lengths [22]. They found that digital scans resulted in significantly smaller measurement depths compared to conventional silicone impressions [22]. In a more recent study that used advanced IOS hardware and software, Leven et al. [23] reported reduced deviation between digital and conventional impressions.

Tomova et al. evaluated CAD/CAM-fabricated posts made from various materials and concluded that digital workflows can be successfully applied to the restoration of severely damaged endodontically treated teeth [24]. They also emphasized that this approach enables the use of a broader range of materials than is feasible with traditional laboratory methods [24]. Similarly, Almaki et al. investigated post cavities of varying depths using different intraoral scanners and CAD/CAM fabrication, and found that this method is a promising alternative to conventional techniques [25].

Studies that have assessed the internal fit and marginal adaptation of CAD/CAM posts fabricated from digital scans have reported results comparable to those obtained through conventional methods, supporting further exploration in this area. Based on these findings, we evaluated five groups to compare scanner efficiency. These included posts fabricated using direct scans with iTero, resin-based impressions scanned with iTero, direct scans with Trios 3, resin-based impressions scanned with Trios 3, and resin-based impressions scanned with the Vinyl extraoral scanner. A control group using a laboratory scanner was also included. The effectiveness of two intraoral scanners, iTero and Trios 3, was assessed in terms of both direct scanning and indirect resin-based workflows.

This study was conducted under laboratory conditions, hence results may not fully reflect clinical conditions. The absence of adjacent tooth structures likely enhanced the scanner’s performance; therefore, different scanning outcomes may occur in actual clinical settings. Furthermore, the study was limited by the number of scanners and post types tested, as well as the use of only one evaluation method. The relatively small sample size also restricts the generalizability of the results to broader clinical scenarios. Future research should investigate a wider variety of post designs, utilize larger sample groups, and incorporate in vivo validation to strengthen and confirm these findings.

To ensure consistency, all groups used standardized teeth, cement, and cavity depth. Resin-based materials were performed using plastic sticks suitable for canal impressions. While some manufacturers offer scanning posts, such as 3Shape’s “Scan Posts,” which are compatible with both direct scanning and plaster model workflows, these were not used due to cost and accessibility constraints. Moreover, these scanning posts have not yet received Food and Drug Administration (FDA) approval for clinical use [26].

The additive manufacturing (AM) process, based on 3D printing technologies, has emerged as a promising production method in the dental industry. Unlike subtractive manufacturing (SM), 3D printing builds the final structure by accumulating successive layers. AM offers several advantages. The most notable is ecological sustainability, achieved through reduced material waste [27]. AM also means lower production costs and greater time efficiency. It enables the fabrication of multiple restorations simultaneously. Recently, manufacturers have introduced three-dimensionally printed composite resins specifically formulated for AM applications in permanent restorations. However, a comprehensive investigation into the mechanical properties of these materials is essential to determine their long-term viability for clinical use.

In this study, an Asiga Max UV 3D printer, which employs the digital light processing (DLP) technique, was used for post fabrication. The posts were produced using Saremco Print Crowntec, a permanent resin crown material. This resin-based material was selected because its elastic modulus is close to that of dentin. Since the polymerization shrinkage of the resin material was taken into consideration, the cement space was set to 0 μm. Before cementation, each post was examined for possible binding spots using a silicone disclosing medium. Öge et al. investigated 30 mandibular teeth divided into two groups [28]. They compared the fracture resistance of teeth restored with 3D-printed resin posts and those restored with fiber posts using a testing machine, and reported no significant difference between the groups. Their results indicated that 3D-printed resin posts performed as efficiently as fiber posts in clinical terms. Similarly, Piangsuk et al. evaluated the accuracy and adaptation of milled CAD/CAM resin post samples, 3D-printed resin post samples, and direct pattern resin post samples [29]. They reported that digitally fabricated posts demonstrated adaptation comparable to that of conventional posts. Prior research on post-and-core systems has reported clinically acceptable cement space values ranging from approximately 50 to 250 μm, with an optimal range often cited between 100 and 300 μm to ensure sufficient retention and reduce the risk of adhesive failure [30, 31]. In the current study, the smallest mean distance was observed in the Vinyl UXD group at the middle mesial region, measuring 66 μm, while the largest mean distance was noted in the Trios ID group at the apical lingual region, at 262 μm. Consistent with prior literature, these measurements fall within the clinically acceptable cement space range for post-and-core restorations.

In this study, micro-CT was used to measure the distance between the post apex and the root canal wall apex in both coronal and sagittal sections. Mean values were compared across all experimental groups. Comparable distances were observed in groups where posts were fabricated via direct intra-canal scanning using iTero Element 2 and Trios 3, as well as in the group utilizing the Vinyl UXD scanner to digitize pattern resin impressions. The greatest apex-to-wall distances were recorded in groups where pattern resin impressions were scanned with iTero and Trios 3, indicating that direct intra-canal scanning produced more accurate depth data. However, no statistically significant differences were found among the groups. Based on micro-CT data, posts fabricated using scan data obtained from Vinyl UXD-scanned pattern resin impressions exhibited the best overall fit. This aligns with previous findings that have identified extraoral scanners as reliable reference systems. Among the intraoral scanners, Trios 3 provided better-fitting posts than iTero Element 2. Nonetheless, direct intra-canal scanning consistently outperformed indirect resin-based scanning in terms of post adaptation accuracy. This finding is consistent with previous studies indicating that the scanning geometry and optical depth of intraoral devices significantly affect adaptation accuracy [25, 29].

Various studies have shown that the accuracy of digital scan data varies significantly between intraoral and extraoral scanners due to differences in optical design, image acquisition strategies, and environmental conditions. Extraoral laboratory scanners, which operate under stable lighting environments and utilize fixed multi-camera systems, generally demonstrate higher precision when capturing conventional impressions or complex geometries. Ender and Mehl found that intraoral scanners may accumulate stitching errors over extended scanning paths, especially in anatomically challenging regions, whereas extraoral systems tend to retain superior trueness [32]. Reviews by Aswani et al. and Nedelcu et al. also observed that intraoral scanners are influenced by optical depth limitations, surface reflectivity, and operator-dependent variability, which can impair accuracy in deep or narrow spaces such as post cavities [33, 34]. Conversely, extraoral scanners, which are not constrained by these clinical factors, provide more stable and reproducible data acquisition. These findings align with the current study, in which the Vinyl UXD extraoral scanner achieved the highest overall internal adaptation, whereas intraoral scanners, although within clinically acceptable ranges, showed greater variability across scanning workflows.

The observed differences between the direct intraoral and indirect resin-pattern workflows are likely attributable to inherent variations in scanner trueness and precision. A comparative study has documented significant variability among devices, with the Trios 3 consistently demonstrating superior performance over several commonly employed intraoral scanners, such as the Medit i700 and Planmeca Emerald, in terms of both trueness and precision [35]. This is attributed to its more advanced optical architecture and reconstruction algorithms [35, 36]. Similar findings have been reported in broader accuracy evaluations, suggesting that scanner performance is predominantly influenced by device-specific characteristics rather than by the scanning modality. Systematic reviews and comparative analyses have indicated that extraoral laboratory scanners tend to produce more stable and reproducible datasets, owing to controlled scanning conditions. In contrast, high-performance intraoral scanners may achieve comparable results depending on the scanning strategy and anatomical complexity [13, 31, 32, 34]. Collectively, these findings underscore the crucial role of scanner-specific optical design, sensor configuration, and software processing in determining the dimensional accuracy of digital scans, particularly in deep or geometrically restricted post spaces, where optical limitations and cumulative deviations are more pronounced.

Micro-CT remains a valuable non-destructive tool for evaluating internal root canal structures and post-cement interfaces in vitro. While alternative techniques such as physical sectioning exist, they carry a risk of specimen damage and are thus less favorable [37]. Numerous studies have demonstrated that micro-CT offers greater accuracy and precision for generating detailed 3D reconstructions of internal canal morphology [38]. For example, Chang et al. [39] used micro-CT to investigate early cement layer dissolution around fiber posts in premolar teeth. Likewise, Soares et al. [40] evaluated the cement–post interface in titanium and glass fiber posts, emphasizing the capacity of micro-CT for precise 3D assessment, especially in hydrated specimens.

## Conclusion

Among the evaluated measurement techniques, the extraoral indirect method, where conventional resin impressions are digitized using an extraoral scanner, yielded posts with superior canal adaptation. Within the limitations of this in vitro study, both direct and indirect digital workflows showed clinically acceptable accuracy after fabrication. Among the tested scanners, intraoral systems performed comparably to the extraoral laboratory scanner. When intraoral digital scanners are selected for post space digitization, direct intracanal scanning is recommended over indirect methods. The additive manufacturing process proved to be a practical method for creating customized posts with good adaptation. Additionally, using permanent resin materials in 3D printing may be a potential alternative for post fabrication; however, further research is needed to confirm their clinical usefulness and long-term durability.

## Data Availability

The datasets generated and analyzed during the current study are available from the corresponding author, Seray Çapar, at caparseray@hotmail.com, on reasonable request.

## Abbreviations

3D: Three-Dimensional
AM: Additive Manufacturing
SM: Subtractive Manufacturing
CAD: Computer-Aided Design
CAM: Computer-Aided Manufacturing
DLP: Digital Light Processing
IOS: Intraoral Scanner
Micro-CT: Micro-Computed Tomography
SLA: Stereolithography
